# Dr. Neill’s Case of Hypertrophy of the Heart and Arteries

**Published:** 1842-05-21

**Authors:** John Neill


### MEDICAL EXAMINER.

NEW SERIES.

No. 21.] PHILADELPHIA, MAY 21, 1842. [Vol. I.

### *Case of Hypertrophy of the Heart and Arteries.*

By John Neill, M. D.

My notes of this case commence with the 2d February, 1842. The patient, Mary Jolly, was a black woman, married, aged 36. She first suffered from pain near her heart 13 years ago. For seven years it gave her little or no trouble, so that she was enabled to pursue her employment of a cook without applying for medical advice. Five or six years ago the pain was often very great, and she perceived "a purring in her throat," to use her own expression. For the last two years she has been unable to work, and is obliged to avoid the least exertion.

When I first saw her she complained of great oppression about her chest, and of difficulty of breathing. Her lungs and bronchi gave no evidence of disease; but percussion over the præcordial region was very flat. The impulses of the heart were slow, heavy and full, the momentum being greater than usual, although the sound was less. Besides the natural sound there was a second, decidedly aneurismal—and upon placing the fingers over the upper portion of the sternum, the thrill was distinctly communicated to the touch. In a recumbent posture the pulsations were evident to the eye. On the right side of the neck, at about the crossing point of the omo-hyoid and sterno-cleido-mastoid muscles, there was also a thrill, leading us to suppose that there might also be an aneurism of the innominata or primitive carotid.

The right brachial artery was so superficial and so much enlarged, that its pulsations were visible in five or six inches of its course. When the fore-arm was flexed it assumed a tortuous appearance near the elbow, where there seemed to be a true aneurism. Its attachments by cellular tissue were so slight, that it could be readily drawn out of its position by the thumb and fore-finger. The left radial and superficial temporal arteries had also this tortuous appearance.

The patient often suffered from attacks of acute carditis, which were temporarily relieved by cups, blisters, and sedative medicines. Her lower extremities were œdematous, and she was liable to constant headache and frequent epistaxis.

A few weeks previous to her death her breathing became so laborious, and her frame so much enfeebled, that she was obliged to keep her bed entirely.

*Autopsy twenty-four hours after death,* in which I was assisted by Drs. Wallace and F. G. Smith.

*General appearance.*—Not much emaciation; face puffy; countenance anxious; eyes prominent.

In laying off the integuments of the throat, we were struck with the enlarged condition of the smaller vessels, and with their very fibrous character. They retained the cylindrical form although empty.

The lungs of each side were perfectly healthy. The pericardium was much enlarged and very thick, but contained a very small quantity of serum, and no lymph. It was adherent to the serous membrane of the heart over a space of more than two inches square—the result of an old inflammation—as it could not be separated until after maceration. On the right side of the right ventricle, the serous membrane had a white and yellow appearance; and the substance of the ventricle itself, a dark purple appearance, giving us the idea that suppuration had commenced. Upon cutting into the yellow spot it was found to be superficial, and the substance of the ventricle firm.

The right auricle was enlarged, so as to be capable of containing half as much more than it usually does. The right ventricle was much enlarged, both in capacity and in the thickness of its sides. The left auricle was enlarged proportionably; but the left ventricle was the seat of the greatest hypertrophy, and it was enormous. Its cavity, walls, columnæ carneæ, mitral and sigmoid valves, gave more the idea of a bullock's heart.

The length of the heart, from its apex to its base, was seven and a quarter inches, and its breadth six inches. The coronary arteries were about the size of a goose-quill, and very fibrous.

In the aorta we certainly expected to find an aneurismal sac, as the throb and murmur had been so distinct in life, but we were mistaken.

The origin of the aorta was in proportion to its ventricle—its diameter was certainly an inch and a half; its coats were several lines thicker than usual, and had the fibrous character mentioned of the inferior thyroid and coronary arteries. At the arch its diameter increased, as might be expected, from the contractile force of such a ventricle; and the passage of the blood through this part gave the thrill and murmur so destinctive of aneurismal sacs.

The innominata was as large as a common sized aorta, and was pushed upwards by the aorta. Its point of bifurcation did not occur in the usual place, but an inch higher up—thus we see how the thrill was produced in this part of the neck. The carotids and their branches bore the same evidences of hypertrophy, not only in diameter, but in length—so that many were tortuous, and all maintained their cylindrical form. The brachial artery, which was so superficial and tortuous, was equally dilated on its whole course, and not only in that portion which resembled an aneurism.

We pursued the examination no further; being convinced that every artery was increased not only in diameter, so as to carry a greater quantity, but also in length, which produced their tortuous appearance, particularly when the muscles were flexed.

